# Identifying NPP operator stressors using grounded theory

**DOI:** 10.1038/s41598-025-16193-0

**Published:** 2025-08-20

**Authors:** Ye Wang, Xiliang Tao, Wenqiang Peng, Zhaopeng Liu, Pengcheng Li

**Affiliations:** 1 School of Nuclear Science and Technology, University of South China, Hengyang, 421001 Hunan China; 2 Key Laboratory of Advanced Nuclear Energy Design and Safety, Ministry of Education, Hengyang, 421001 China; 3 Taishan Nuclear Power Joint Venture Co., Ltd, Taishan, 529200 Guangdong China; 4 State Key Laboratory of Nuclear Power Safety Technology and Equipment, China Nuclear Power Engineering Co., Ltd, Shenzhen, 518172 Guangdong China; 5 Human Factor Institute, University of South China, Hengyang, 421001 Hunan China

**Keywords:** Nuclear power plant operators, Psychological stress, Grounded theory, Multidimensional load, Health care, Health occupations

## Abstract

Nuclear power plant (NPP) operators manage crucial decision-making and operational tasks in precarious and complex environments, where psychological stress directly correlates with system safety and operational efficiency. However, the existing research largely explores single factors or specific scenarios, lacking systematic analysis. This study aims to identify psychological stress-influencing factors for nuclear power plant operators based on grounded theory. Accordingly, work experience data were collected from 25 operators in NPPs through semi-structured interviews. Using open, axial, and selective coding, we identified key categories and constructed a multidimensional load model of psychological stress. The finding revealed that operators’ psychological stress was attributable to the following seven factors: interface management load, task complexity, alarm load, transient and emergency task load, shift work and continuous load, error handling load, and communication load. Moreover, the stressors interact and compound, elevating operators’ psychological and cognitive burdens. Overall, this study presents a novel outlook for comprehending psychological stress in precarious environments and recommends further investigation of the dynamic correlations among these factors.

**Supplementary Information:**

The online version contains supplementary material available at 10.1038/s41598-025-16193-0.

## Introduction

In complicated industrial systems, NPP operators shoulder the crucial responsibility of supervising and regulating nuclear reactors, working in a high-risk and complex environment^1^. Owing to the prerequisite for swift decision-making and response to emergencies under high pressure over a prolonged term, the psychological stress experienced by operators has garnered considerable attention. Pertinent research has demonstrated that the proportion of errors due to psychological stress is as high as 47.48% in human-related incidents reported in nuclear power plants^1^. Mumaw categorized the stressors for nuclear power plant operators into environmental factors, novelty events, and task-related factors, as well as proposed pathways to augment operator performance^2^. Cox and Cox further refined the stressors, recognizing the main factors influencing operators, such as workload, uncertain events, time pressure, automation interference, noise, team collaboration, and communication issues^3^. In China, Jiang et al. highlighted that the psychological burden on NPP operators is largely depicted in task difficulty, mental and physical demands, tension, and fatigue^4^. Furthermore, Sun et al. demonstrated that sustained time pressure not only influences operators’ diagnostic and monitoring skills but could also endanger the safety of the entire nuclear power plant system^5^.

Initial research into operators’ psychological stress mainly concentrated on pinpointing fixed or unchanging sources of stress. For example, studies explored how the complexity of interfaces in digital control rooms affects the cognitive load experienced by operators. It was discovered that intricate interface designs increased the demands of information processing, which in turn raised operators’ cognitive load^6–8^. This method generally took a human-machine interaction perspective, assessing possible psychological stress impacts by analyzing factors such as control room layout, information density, and the complexity of visual displays.

According to Xu et al., conventional Human Reliability Analysis (HRA) methods like SPAR-H recognize stress-related Performance Shaping Factors (PSFs) such as ‘complexity’ and ‘time availability,’ but largely depend on subjective evaluations by experts. As a result, these approaches face challenges in accurately reflecting the cumulative effects of stress that emerge from ongoing dynamic interactions^9^.

With the advancement of research, scholars have come to acknowledge that psychological stress unfolds as a dynamic process. As a result, research focus gradually moved away from static observations toward examining the underlying dynamic mechanisms. For example, recent studies started examining how alarm load correlates with variations in task execution pathways. In complex systems such as nuclear power plants, frequent alarms and false alerts have a substantial impact on the psychological state of operators. A high volume of alarms forces operators to handle extensive amounts of information within short periods. This increases psychological strain and can potentially disrupt task execution pathways, which may undermine both system safety and operational effectiveness.

Recent studies on operators’ psychological stress have progressed toward a more multidimensional and in-depth understanding. Utilizing neuroergonomic approaches has offered new perspectives on psychophysiological reactions under stress. For example, Braarud’s comprehensive review showed that alarm load, such as frequent or false alarms, intensifies stress via dual pathways. Through the cognitive pathway, this is evidenced by increased theta-wave activity in the prefrontal cortex, indicating decision-making conflicts during operations^10^. On the behavioral side, it appears as diminished visual search efficiency, marked by lower blink frequency and enlarged pupil diameter. These physiological shifts are associated with a 20% rise in the likelihood of missing critical signals. This multidimensional approach allows for a thorough evaluation of psychological stress effects and offers practical insights for improving human-machine interface design.

At the same time, more detailed analyses have been conducted on the long-term impact of shift work on circadian rhythms and fatigue buildup, as well as their association with operational safety. Prolonged shift work disrupts operators’ biological rhythms, precipitating cumulative fatigue. Such fatigue diminishes cognitive performance and slows reaction times, increasing the likelihood of errors during crucial tasks. Recent research emphasizes reducing these negative impacts by improving shift schedules and adapting the work environment to boost both efficiency and safety^11,12^.

While stressors and their effects on NPP operators have been explored comprehensively, the existing research mainly focused on specific scenarios or single factors, lacking a systematic and integrative analysis. To thoroughly investigate the intricate causes and fundamental associations behind operators’ psychological stress and to build a unified theoretical model from the bottom up, this study employs grounded theory methodology. Based on interview data and literature analysis, we investigate the formation and influencing factors of psychological stress among NPP operators, constructing a multidimensional model of these influencing factors.

## Methods

Grounded theory is particularly effective for exploratory studies, especially when examining complex influencing factors in areas lacking well-established theoretical frameworks. Rajabi et al.used open, axial, and selective coding on 49 unique workplace safety risk management (WSRM) errors, organizing them into seven phase-specific categories, with ‘risk identification failure’ identified as the central theme^13^. Through interviews with 26 individuals diagnosed with bipolar disorder, Pereira et al. recognized ‘acceptance of diagnosis’ as the core category^14^. Zhang et al. conducted a three-tier coding analysis of 60 literature sources and 23 stakeholder interviews, which uncovered three key dimensions, natural, economic, and social, within urban innovation processes^15^. In this framework, ‘economic drive’ emerged as the central category because of its strong causal impact. Liu et al. conducted a grounded theory-based systematic review of 230 studies on safety behavior among frontline workers, synthesizing thousands of insights into a structured four-level framework: individual competence → managerial behavior → organizational climate → work context^16^. Similarly, Gu & Lu and Karimi et al. distilled 181 initial nodes related to educational satisfaction and 19 key factors contributing to unsafe behavior in automotive safety into seven and four main categories, respectively^17,18^.

Thus, grounded theory reliably brings together diverse influencing factors into unified, higher-level categories, whether in the context of mental health recovery, urban governance, or industrial safety. This serves as a crucial conceptual basis for later quantitative validation and the design of focused interventions.

**Fig. 1:**
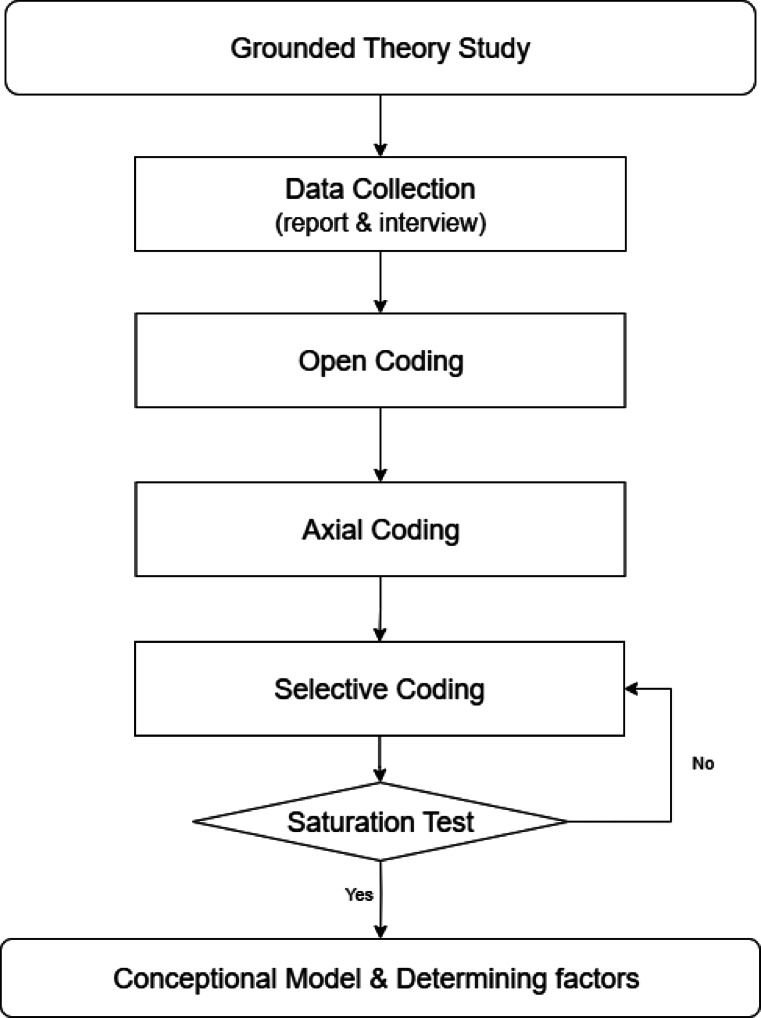
Research Steps of Grounded Theory

Through comprehensive interviews with nuclear power plant operators, potential stressors and their underlying impacts were identified to conceptualize a preliminary theoretical framework. This process involved open coding, axial coding, selective coding, and theoretical saturation assessment. The authors affirm that all procedures were conducted in full compliance with national ethical standards, institutional protocols, and applicable guidelines. The methodology is illustrated in Fig. 1.

### Interview guide design

Before finalizing the interview guide, 3 experts with extensive experience in the management and training of NPP operators were invited to join discussions. The guide was refined through multiple rounds of revision by aligning the Job Demands-Resources (JD-R) model with practical insights from industry. Originally designed to explain the etiology of occupational stress, the JD-R model exemplifies a specific application of the Conservation of Resources (COR) theory in organizational settings^19^. The experts advised focusing on factors like interface complexity, task complexity, emergency response, and team collaboration.

### Data collection

This study explores the factors contributing to psychological stress among NPP operators by using a qualitative approach, incorporating semi-structured interviews to thoroughly examine their work experiences and sources of stress. The participants consisted of currently employed frontline operators working at domestic NPPs equipped with EPR and AP1000 reactor technologies. The interviews were carried out in two phases, first in December 2024 and then in July 2025. Twenty-five operators took part in the study, occupying roles such as Shift Supervisor, Deputy Shift Supervisor, Isolation Coordinator, Senior Control Room Operator, and Control Room Operator. On average, participants had 12.4 years of work experience. All interviews were conducted face-to-face in a one-on-one format, lasting 30–40 min each to avoid interrupting the participants’ standard work. Before the interviews, all participants signed informed consent forms, confirming that data collection strictly complied with ethical requirements and that they had the right to stop the interview at any time. During the interviews, nondirective questioning was used to let the participants freely express their true experiences and feelings. Moreover, probing techniques were used to further assess potential stressors. For instance, when participants mentioned “equipment failure,” interviewers asked follow-up questions about its detailed impact on their psychological state, coping strategies, and recovery processes.

All interview contents were recorded and transcribed into text instantly after the interviews to safeguard the completeness and accuracy of the information. A total of over 180,000 words of interview records were collected, and the textual data were proofread three times to confirm uniformity with the respondents’ original intentions and to evade any omission or bias. During the data-processing stage, anonymization was applied by assigning codes (e.g., O1 to O25) to the respondents to guarantee that individual information could not be directly identified, further protecting respondent privacy and data security.

## Results

### Open coding

**Fig. 2:**
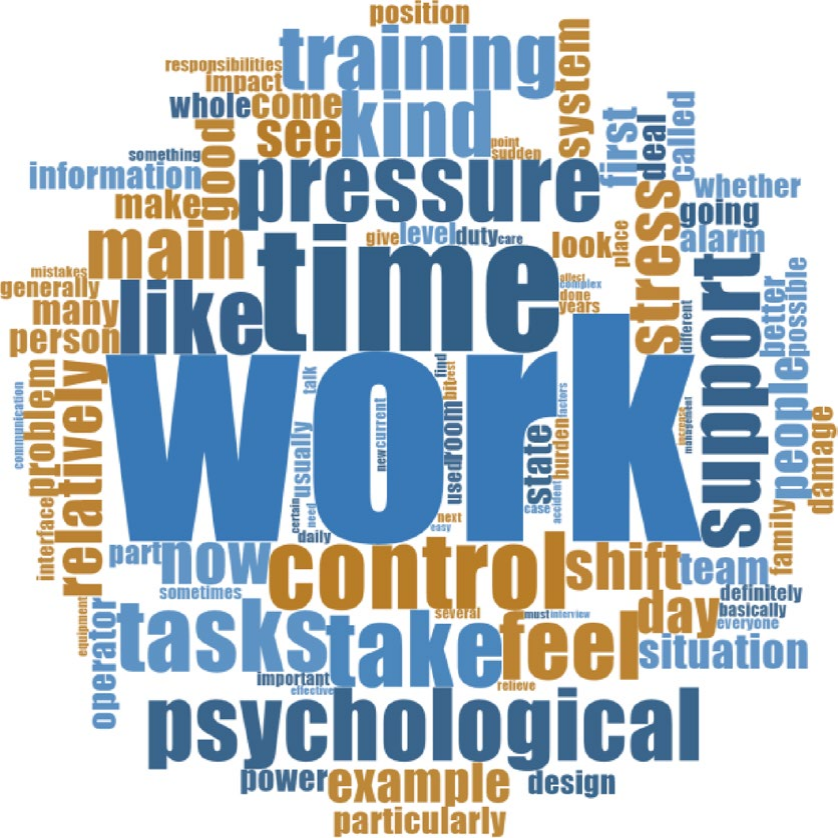
Word Cloud

In grounded theory research, open coding serves as the first stage of analysis, focusing on identifying and deriving conceptual categories from the raw data. In this study, a thorough line-by-line examination of the in-depth interview data was carried out to identify conceptually meaningful statements and develop initial categories. To maintain data authenticity and reduce subjective bias, the respondents’ exact words were used when recording the data. Further, NVivo 12 Plus software was used to improve the precision and efficiency of the coding process, therefore reinforcing the rigor of data analysis. During the analysis, we remained aligned with the research objectives, thoroughly reviewed participants’ responses, and continually refined relevant categories and concepts. We coded three-quarters of the sample data, that is, 19 interview records, and reserved the remaining 6 for saturation testing. Given the complexity and redundancy of the initial concepts, we categorized them and eliminated those with low frequency or inconsistent expressions. After repeated analysis of open coding, a total of 94 initial concepts were ultimately identified. This process ensured the accuracy and reliability of the research findings. The results of open coding are detailed in *Appendix A*, with word frequency statistics presented in Table 1 and word cloud Fig. 2.

**Table 1:** Word Frequency.

Word	Length	Count	Weighted Percentage (%)
work	4	489	2.13
time	4	409	1.78
pressure	8	260	1.13
psychological	13	253	1.10
kind	4	227	0.99
support	7	149	0.65
tasks	5	139	0.61
team	4	138	0.60
control	7	136	0.59
example	7	129	0.56
training	8	123	0.54
shift	5	115	0.50
system	6	112	0.49
whether	7	112	0.49
good	4	104	0.45
stress	6	104	0.45
take	4	101	0.44
task	4	101	0.44
information	11	94	0.41
relatively	10	89	0.39
see	3	89	0.39
state	5	86	0.38
make	4	85	0.37
need	4	85	0.37
definitely	10	82	0.36
room	4	81	0.35
alarm	5	79	0.34
operator	8	79	0.34
problem	7	77	0.34
something	9	77	0.34
interface	9	76	0.33
everyone	8	75	0.33
daily	5	74	0.32
deal	4	74	0.32

### Axial coding

Axial coding, the second level of coding in grounded theory, explores and establishes correlations between concepts and categories. The extracted conceptual categories are often superficial during the open coding phase. Therefore, axial coding further simplifies and summarizes these initial categories by examining the causal and structural correlations among concepts, constructing correlations between nodes. A higher node count indicates that the phenomenon or feature appears more frequently in the data, reflecting a higher level of attention from the participants. This stage encompasses merging keywords with consistent or similar expressions into higher-level categories and constructing logical associations to finally create main categories. In this study, the initial concepts obtained from open coding were systematically summarized to finally extract the following seven main categories: interface management load, task complexity, alarm load, transient and emergency task load, shift and sustained load, error-handling load, and communication load (Table 2).

**Table 2:** Axial coding process.

Core Categories	Node Count	Subcategories	Node Count	Connotation
**Interface Management Load**	27	High Information Density in User Interface	8	Excessive interface elements with dense arrangement, containing overloaded information/controls
		Complex Layout	7	Critical parameters buried in deep menu layers, requiring multistep navigation
		Operational Complexity	12	Frequent interface switching increases task execution difficulty
**Task Complexity**	52	Multitasking	15	Parallel task execution causes cognitive resource competition
		Procedural Complexity	10	Task sequences with more independent steps requiring working memory retention
		Safety-Critical Tasks	18	Risk-significant actions requiring precise execution per technical specifications
		Cognitive Demand	9	Tasks involving diagnostic reasoning or complex decision-making
**Alarm Load**	29	Alarm Frequency	11	High-rate alarm causing information overload
		High-Volume Alarm Activation	13	Multiclass alarm systems requiring prioritization under time pressure
		Nuisance Alarms	5	Spurious/nonconsequential alarms reducing operator trust
**Transient & Emergency Load**	36	Emergency Response	14	Immediate action required for unanticipated events
		Accident Management	16	Post-accident scenario requiring simultaneous execution of EOPs/SAMGs
		Novel Event Handling	6	First-of-a-kind events requiring non-proceduralized decision-making
**Shift & Sustained Load**	33	Night Shift Effects	7	Circadian rhythm disruption affecting vigilance
		Sustained Task Volume	17	Extended task duration causing performance degradation
		Shift Rotation Impact	9	Frequent shift changes disrupting sleep patterns
**Error Handling Load**	18	Error Documentation	4	Post-error procedural requirements
		Error Analysis Burden	8	Root cause analysis per ASME/ANS RA-S-1.4 standards
		Error Recovery	6	Compensatory actions requiring extended system monitoring
**Communication Load**	16	Team Coordination Issues	5	Delayed information transfer violating NRC communication standards
		Role Ambiguity	7	Unclear responsibility allocation contradicting the station’s SAT program requirements
		Information Asymmetry	4	Incomplete data sharing affecting situation awareness

### Selective coding

Selective coding is the third level of coding in grounded theory, aimed at forming relationships between categories. This process entails continuous comparison, analysis, and refinement of the core concepts, exploring how the main categories are interrelated, identifying their logical connections, and ultimately constructing a comprehensive view of behavioral dynamics to develop a complete theoretical framework. Our findings indicate that these categories can be further distilled into a single core category: “Factors Influencing Psychological Stress of NPP Operators”. The influencing factors mainly include “Interface Management Load (A1),” “Task Complexity (A2),” “Alarm Load (A3),” “Transient and Emergency Task Load (A4),” “Shift and Sustained Load (A5),” “Error Handing Load (A6),” and “Communication Load (A7).” Based on interview narratives, the following relationships were established among these categories. Therefore, the outcomes of the selective coding process in this study are presented in Table 3.

**Table 3:** Typical relationship structure of main Categories.

Typical Relationship Structure	Connotation of the Relationship Structure
Interface Management Load → Cognitive Load:	Complex interface designs cause operators to expend excessive cognitive resources on information retrieval and task execution, leading to attention diversion and operational delays, increasing psychological stress.
Task Complexity → Decision-making Pressure	The characteristics of parallel tasks, high-risk operations, and complex procedural steps require operators to make high-precision decisions within limited time frames, resulting in increased psychological tension and the risk of errors.
Alarm Load → Information Overload	High-frequency alarms, false alarms, and disorganized classification create pressure on operators to filter information, potentially causing critical signals to be overlooked and interfering with primary task execution, triggering anxiety in emergencies.
Transient and Emergency Task Load → Emergency Overload	The unpredictability and urgency of sudden tasks force operators to rapidly switch task modes, resulting in short-term cognitive overload, which exacerbates psychological tension and operational uncertainty.
Shift and Long-term Task Load → Fatigue Accumulation	Night shifts, frequent shift changes, and prolonged repetitive tasks lead to disruptions in circadian rhythms and decreased attention, with accumulated fatigue further reducing operational efficiency and creating a vicious cycle of psychological stress and physical depletion.
Operational Error Load → Remediation Pressure	Post-error debriefing, remediation, and documentation processes add extra workload, while also causing feelings of frustration and performance anxiety, forming a negative feedback loop of “error-stress-reerror.”
Communication and Collaboration Load → Team Friction	Delays in information transmission, ambiguous role allocation, and negative communication behaviors reduce team collaboration efficiency. Friction during task execution further distracts individual attention and amplifies overall psychological load.

### Theoretical saturation test

**Fig. 3:**
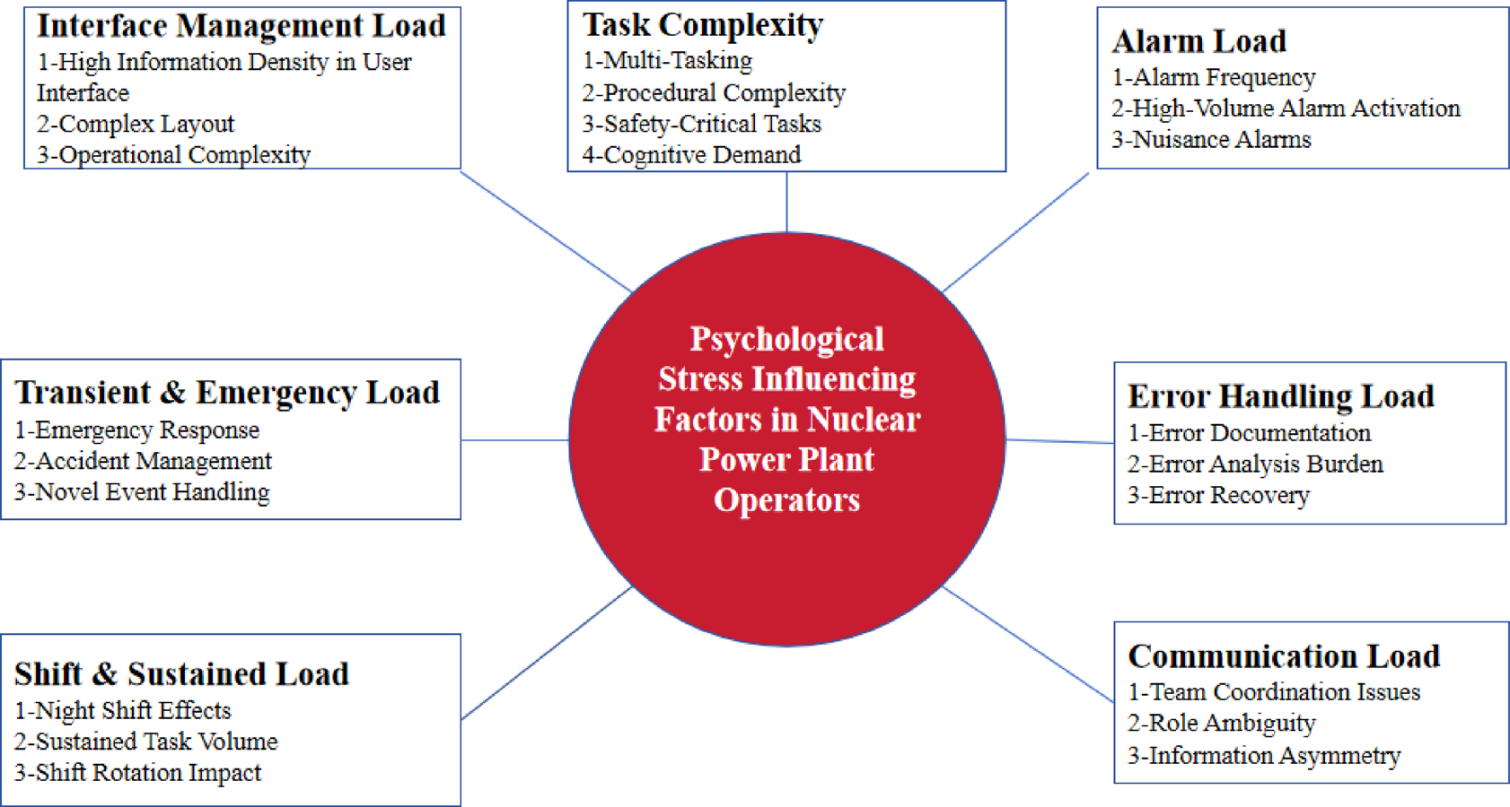
Theoretical Model Diagram of Influencing Factors

To authenticate the reliability and validity of the study and ensure that the developed theory had reached saturation, a saturation test was performed by the authors. The remaining six interview records were coded, and no new significant categories were identified, indicating that saturation had been achieved and that sampling could be discontinued. Figure 3 illustrates the model of influencing factors developed in this study.

## Discussion

### Interface management load

In an NPP setting, interface management load is a leading cause of operators’ psychological stress. This mainly includes the cognitive burden imposed by complex human–machine interaction interfaces and frequent interface switching. Recent ERP findings suggest that interface complexity, quantified by image entropy, has an optimal range between 1.28 and 1.79; going beyond this threshold significantly reduces decision-making accuracy and prolongs reaction time as a result of cognitive overload^20^. The digital instrument panel interface is complicated, with several elements closely arranged and critical data often nested within deep layers of the interface; this pressurizes operators to spend extra time searching for target information. Moreover, recurrent switching between multiple layers of interfaces during task execution makes it challenging for operators to maintain focus, elevating the chances of operational errors. Similarly, the complex interface layout and extensive data input and output mandate operators to filter large volumes of information quickly, which can cause overlooking of crucial information. Such highly intricate interfaces warrant substantial cognitive resources from operators, and prolonged intense concentration can result in psychological stress build-up, corroborating some previous studies^6–8^. Thus, the issues mentioned above can be addressed by streamlining the human–computer interface design, simplifying the operation process, and creating task-specific interfaces, effectively reducing operators’ interface management burden. This will alleviate operators’ psychological stress and enhance the safety and stability of the nuclear power plant.

### Task complexity

Task complexity is a key factor influencing operators’ psychological load and operational performance. Complex tasks are frequently multidimensional, such as parallel subtasks, numerous steps, high operational risks, and substantial cognitive demands. Recent studies employ a dual-perspective framework, combining Halstead’s E measure for dynamic execution and entropy metrics for static structure, to quantify this, showing that Task Execution Complexity (TEC) and Task Information Complexity (TIC) directly contribute to increased cognitive load^21^. These attributes pose considerable challenges to operators’ attention allocation, information-processing skills, and decision-making speed. A comparative analysis of the existing literature revealed that task complexity not only directly affects the reliability of operators’ actions but also significantly elevates psychological stress and the probability of errors in emergencies.

The TACOM model, proposed by Park and Jung and highly referenced for the quantitative assessment of task complexity, classifies task complexity into five dimensions: information volume, number of operations, logical entanglement, domain knowledge requirements, and engineering decision-making demands^22^. This framework closely aligns with the multidimensional definition of task complexity in this study, both highlighting the multilevel impact of complex tasks on operators’ psychological states and performance. Although TACOM emphasizes immediate operational effects, the integrated model by Pang and Dai extends this understanding by capturing the accumulated psychological strain, demonstrated through strong correlations between TEC and both NASA-TLX workload scores (R^2^ = 0.679) and execution time (R^2^ = 0.785), especially during high-risk parallel tasks^21^. However, while the TACOM model mainly focuses on the direct impact of task complexity on operation time and error rates, this study further emphasizes the cumulative effect of complex tasks on operators’ psychological burden, accentuating the significance of parallel tasks and high-risk tasks in actual operational situations.

### Alarm load

In the complex main control room environment, high-frequency, multicategory, and false alarms significantly surge the information-processing burden on operators, adversely affecting their attention allocation and task execution speed. Frequent and complex alarm systems can result in information overload, making it difficult for operators to filter vital information timely manner, therefore affecting operational decisions and system safety. Park et al. highlighted that the complexity of alarm systems directly impacts operators’ performance in emergency tasks. Further, frequent false alarms and alarm interference in multitask environments can cause operators to overlook critical alarms or make operational errors because of unsuitable information filtering^23^. Kim et al. further established the correlation between alarm load and task execution path variability, demonstrating that the higher the alarm load, the higher the variability in task execution paths^24^; that is, operators under high alarm load are more likely to adopt different strategies to handle complex situations. A heavy alarm load, particularly when false alarms are present, significantly hinders the ability to recognize genuinely critical alerts. Operators must exert greater effort to assess the validity and urgency of signals. This uncertainty in decision-making can lead to increased anxiety and stress.

### Transient and emergency task load

The operation of a nuclear power plant entails transient phenomena, which are complex processes caused by internal/external factors, creating a short-term deviation of the system state from its standard steady state. Typically, these phenomena are displayed as rapid deviations in reactor power, cooling system failures, or sudden leaks^25^. Meanwhile, transients are depicted by rapid fluctuations in system parameters and compound decision-making requirements, posing a critical challenge for nuclear power plant operators. Reportedly, the system instability and the reaction of multiple variables during transients surge the cognitive load and psychological stress on operators^26^. For instance, when a coolant leak or a rupture occurs in the steam generator piping, operators must swiftly evaluate the equipment status, sort information, and react to the situation, for improper handling can worsen the accident^5^. The unpredictability and novelty of emergency tasks further amplify operators’ difficulty in handling the situation^2,27^. Furthermore, the dynamic and complex nature of nuclear power plant operations warrants operators to have rapid response skills and high concentration. However, frequent shift work and night shifts can reduce their attention span, raising the variability and risk of errors in transient task execution.

### Shift work and long-term task load

In high-risk industries, such as nuclear power plants, shift work and long-term duty exert multifaceted and profound effects on operators’ physical and mental health and their job performance. Nesthus et al. reported that night shifts can cause sleep deprivation and phase delays in circadian rhythms, therefore recommending a clockwise shift rotation to decrease circadian rhythm disruption^11^. This study further underscores that circadian rhythm disruption not only affects operators’ emergency response skills but also weakens their analytical ability during routine operations, particularly in settings like nuclear power plants that warrant high concentration and precise operations.

McGuffog et al. highlighted that the risk of fatigue amplifies significantly after > 6 h of continuous work, which aligns with our study^12^. Unlike general industries, the complexity of tasks and high demand for precise operations in nuclear power plants further emphasize the impact of fatigue. This condition is often aggravated by the continuous rise in task volume and extensive business training, putting additional psychological burdens on operators even before they recover from physical fatigue. Besides, the buildup of long-term tasks lowers operators’ sensitivity to anomalous signals and increases the probability of miscalculating system states. Furthermore, excessive cognitive load can easily cause psychological fatigue and occupational burnout, further decreasing work efficiency and significantly increasing risk exposure during crucial moments.

### Operational error load

In this study, operational error load is defined as the psychological pressure and additional cognitive demands caused by task execution failures. After an error occurs, operators must engage in rigorous debriefing and remediation processes, which substantially elevate their psychological and cognitive burdens. Moreover, the recording and analysis of errors often increase anxiety, further weakening operators’ focus and critical thinking aptitude in subsequent tasks. Further, external reviews and remediation work multiply the task load, deplete energy and time resources, and render operators more susceptible to judgment errors or delayed reactions in subsequent operations. Hancock highlighted that failed experiences are a major source of workload in system operations, and the perceived workload from failures is significantly higher than that from successes^28^; this view aligns with practices in nuclear power plant environments. Thus, managing operational error load is crucial, especially in high-risk industries where it is imperative to mitigate the adverse impacts of errors through optimized operational procedures and supportive interventions.

### Communication and collaboration load

The communication and collaboration load denotes the additional work burden perceived by team members because of poor communication or ineffective collaboration during information transmission and collaborative processes. This type of load adversely affects task execution efficiency and team performance. In complex operational environments, asymmetric or delayed information can prevent operators from obtaining comprehensive information at critical moments, raising the risk of misjudgment and operational errors. Kauffeld et al. suggested that negative communication behaviors, such as complaining and criticizing, can elevate individual stress and fatigue, underlining the close correlation between communication quality and psychological load^29^. Maynard et al. further demonstrated that informal communication methods could result in higher perceived workloads among team members, establishing the potential impact of communication patterns on load perception^30^. Moreover, Lehmann-Willenbrock illustrated that in high-pressure settings, problem-oriented communication, although potentially improving task focus, can also elevate the perceived load on team members^31^. All these studies theoretically support the findings of this study, exemplifying a significant correlation between communication quality and team members’ psychological load and task performance in high-risk settings like nuclear power plant operations. Thus, optimizing communication methods and augmenting information transmission efficiency are key strategies for decreasing overall load and enhancing system safety.

### Limitations

Though this work makes a multidimensional workload model of psychological stress among NPP operators, several limitations warrant acknowledgment. Firstly, the sample size was limited, as all participants were recruited from only two domestic NPP sites. This narrow geographic and institutional scope may restrict the applicability of the findings to wider operational contexts. Second, while the research examined the independent effects of individual stressors, it did not explore the interconnections among stressors or the dynamic mechanisms through which they interact. Elucidating these synergistic effects represents a critical avenue for future research.

## Conclusions

This study establishes that nuclear power plant operators’ psychological stress primarily stems from the following seven factors: interface management load, task complexity, alarm load, transient and emergency task load, shift work and long-term task load, task failure load, and communication and collaboration load. These stressors interact in the high-risk and intricate work environment, aggravating operators’ psychological and cognitive burdens. Overall, this study offers a novel theoretical basis for comprehending the causes of psychological stress in high-load work settings.

## Supplementary Information

Below is the link to the electronic supplementary material.

Supplementary Material 1

## Data Availability

The datasets used and/or analysed during the current study available from the corresponding author on reasonable request.
