# Supplementary material for: Identifying NPP operator stressors using grounded theory
Source: Sci Rep. 2025 Aug 20;15:30618. doi: 10.1038/s41598-025-16193-0 (PMC12368191; doi:10.1038/s41598-025-16193-0)
Supplement: Supplementary file 1 — Supplementary Material 1 [file 41598_2025_16193_MOESM1_ESM.docx]

**Appendix A. Open coding analysis of interviews.**

| **Examples of Interview Text** | **Conceptualization** | **Initial Concepts** |
| --- | --- | --- |
| “Frequent navigation tasks in the main control room consume significant time due to interface switching.” | Multiple interface switching increases cognitive load | A01 Complex Navigation Tasks A02 Frequent Interface Switching A03 Complicated Operation Procedures |
| “Need to monitor multiple screens simultaneously, leading to attention diversion.” | Multiscreen monitoring tasks cause attention diversion | A04 Multiscreen Monitoring A05 Attention Diversion A06 Multitasking A07 Increased Visual Burden |
| “Time-consuming equipment status retrieval in complex interfaces increases task load.” | Complex interface designs hinder information retrieval | A08 Time-Consuming Equipment Status Search A09 Complex Interface Operation A10 Prolonged Task Execution A11 Elevated Operational Burden |
| “Frequent interface layout adjustments are required for critical data visualization, extending task duration.” | Dynamic interface layout adjustments reduce task efficiency | A12 Frequent Layout Adjustments A13 Hidden Critical Data A14 Extended Task Duration A15 Cumbersome Operational Procedures |
| “Reset tasks require multilayer interface navigation, delaying fault handling.” | Multilayer interface reset processes impede fault recovery | A16 Complicated Reset Tasks A17 Multilayer Interface Navigation A18 Delayed Fault Handling A19 Complex Operational Pathways |
| “Lack of shortcuts in certain interfaces necessitates hierarchical access for operations.” | Hierarchical interface layers increase operational complexity | A20 Absence of Shortcut Functions A21 Hierarchical Access Operations A22 Complex Interface Navigation A23 Time-Consuming Operations |
| “Lengthy comprehension of complex regulations elevates task pressure.” | Complex regulatory frameworks reduce task efficiency | A24 Complexity of Regulatory Comprehension A25 Time-Consuming Regulatory Review A26 Increased Task Pressure A27 High Information Volume |
| “Parallel task execution disrupts focus on primary operations.” | Parallel task management interferes with core task performance | A28 Multitasking Requirements A29 Attention Fragmentation A30 Difficulty in Single-Task Focus A31 Reduced Operational Efficiency |
| “Handling multiple system failures simultaneously increases coordination complexity.” | Simultaneous multisystem task execution elevates the psychological burden | A32 Simultaneous Handling of Multiple System Failures  A33 Difficulty in personnel coordination A34 High Resource-Allocation Pressure |
| “When performing high-risk tasks, multiple confirmations are needed as every detail must be correct.” | High-risk tasks require precise operations, increasing tension | A35 High-Risk Tasks A36 Multiple-Confirmation Process  A37 Strict Detail Checking A38 Low Error Tolerance Rate |
| “New task operations require extended time for learning and familiarization.” | Inexperience with new tasks prolongs execution time | A39 Extended Learning Period for New Tasks A40 Operational Unfamiliarity A41 Prolonged Adaptation Phase A42 Heightened Training Requirements |
| “Novel events frequently trigger operational errors.” | Novelty events amplify cognitive load | A43 Unfamiliar Novel Events A44 Error-Prone Operations A45 Insufficient Experience Accumulation A46 Elevated Emergency Response Difficulty |
| “A feedwater loss event triggers high-volume alarms, demanding urgent response under extreme pressure.”​ | Sudden alarm surges impose emergency processing and cognitive overload | A47 High-Volume Alarm Activation A48 Acute Response Pressure A49 Concentrated Task-Processing Demand A50 Aggravated Psychological Burden |
| “Ambiguous alarm classification risks critical alarm oversight.” | Disorganized alarm classification complicates prioritization | A51 Ambiguous Alarm Classification A52 Cover Critical Alarm  A53 Conflicting Information Prioritization A54 Key Event Oversight Vulnerability |
| “Persistent alarms disrupt operator focus, inducing irritability.” | Continuous auditory alarms interfere with task execution | A55 Persistent Auditory Alarm Disturbance A56 Attention Fragmentation A57 Operator Irritability A58 Reduced Task Focus |
| “Unplanned events demand immediate response, causing acute stress within limited timeframes.” | Unplanned tasks intensify time pressure perception | A59 Immediate Response to Unplanned Events A60 Acute Stress Perception A61 Time-Constrained Operations A62 Anxiety in Emergency States |
| “Transient events require immediate operator intervention, escalating emergency response burden.”​ | Task surges under emergencies induce operator overload | A63 Transient Event Emergence A64 Operator Immediate Intervention Requirement  A65 Emergency Response Overload  A66 Escalated Operational Burden |
| “A sudden 20% load rejection necessitates rapid system stabilization under extreme time pressure.” | Load rejection incidents impose urgent stabilization demands | A67 Load Rejection Incident A68 Urgent System Stabilization Demand A69 Intensified Operational Stress A70 Critical Response Time Constraint |
| “Extended shifts degrade operator attentiveness, elevating error risks.” | Shiftwork and sustained high-intensity tasks induce fatigue | A71 Extended Shift Duration A72 Degraded Attentional Vigilance A73 Increased Error Probability A74 Operator Fatigue Accumulation |
| “Prolonged repetitive tasks lead to operator burnout.” | Repetitive tasks induce cognitive fatigue | A75 High-Task Repetitiveness A76 Operator Burnout Syndrome A77 Reduced Work Engagement A78 Mental Fatigue Progression |
| “Post-error task re-execution amplifies psychological stress.” | Repetitive operations post-error exacerbate psychological stress | A79 Post-Error Task Re-Execution A80 Repetitive Procedure Implementation A81 Enhanced Frustration Perception A82 Amplified Psychological Load |
| “Formal documentation of errors may escalate operator psychological burden.” | Error records cast psychological shadows on subsequent tasks | A83 Formal Error Documentation Protocol A84 Progressive Stress Escalation A85 Performance Anxiety Induction  A86 Error-Averse Behavioral Tendency |
| “Ineffective communication among operators during incidents causes task delays.” | Poor team communication impairs task execution | A87 Ineffective Interoperator Communication A88 Task Timeline Disruption A89 Suboptimal Team Coordination A90 Delayed Information Dissemination |
| “Uncoordinated multiperson task execution increases failure risks.” | Dysfunctional teamwork elevates task failure probability | A91 Teamwork Incompatibility  A92 Multiperson Task Conflict A93 Poor Operational Synchronization A94 Risk of Coordinated Task Failure |
